# A DNA Barcode Library for North American Pyraustinae (Lepidoptera: Pyraloidea: Crambidae)

**DOI:** 10.1371/journal.pone.0161449

**Published:** 2016-10-13

**Authors:** Zhaofu Yang, Jean-François Landry, Paul D. N. Hebert

**Affiliations:** 1 Key laboratory of Plant Protection Resources and Pest Management, Ministry of Education, Northwest A&F University, Yangling, Shaanxi, China; 2 College of Plant Protection, Northwest A&F University, Yangling, Shaanxi, China; 3 Agriculture and Agri-Food Canada, Ottawa Research & Development Centre, Ottawa, Ontario, Canada; 4 Centre for Biodiversity Genomics, Biodiversity Institute of Ontario, University of Guelph, Guelph, Ontario, Canada; National Center for Biotechnology Information, UNITED STATES

## Abstract

Although members of the crambid subfamily Pyraustinae are frequently important crop pests, their identification is often difficult because many species lack conspicuous diagnostic morphological characters. DNA barcoding employs sequence diversity in a short standardized gene region to facilitate specimen identifications and species discovery. This study provides a DNA barcode reference library for North American pyraustines based upon the analysis of 1589 sequences recovered from 137 nominal species, 87% of the fauna. Data from 125 species were barcode compliant (>500bp, <1% n), and 99 of these taxa formed a distinct cluster that was assigned to a single BIN. The other 26 species were assigned to 56 BINs, reflecting frequent cases of deep intraspecific sequence divergence and a few instances of barcode sharing, creating a total of 155 BINs. Two systems for OTU designation, ABGD and BIN, were examined to check the correspondence between current taxonomy and sequence clusters. The BIN system performed better than ABGD in delimiting closely related species, while OTU counts with ABGD were influenced by the value employed for relative gap width. Different species with low or no interspecific divergence may represent cases of unrecognized synonymy, whereas those with high intraspecific divergence require further taxonomic scrutiny as they may involve cryptic diversity. The barcode library developed in this study will also help to advance understanding of relationships among species of Pyraustinae.

## Introduction

DNA barcoding [1, 2] is now generally accepted as an effective tool for rapid, accurate species-level identifications and for the discovery of cryptic species across the animal kingdom [3–5]. Several studies have demonstrated over 90% discrimination of species in comprehensive studies on Lepidoptera [6–8]. Morever, it has helped to resolve taxonomic problems and to advance understanding of speciation patterns [9, 10].

The Pyraustinae is the third largest subfamily (after Spilomelinae and Crambinae) in the Crambidae with about 190 genera that include over 1400 described species, approximately 14.6% of all crambid species [11, 12]. Their larvae feed mainly on the stems and fruits of herbaceous plants and include many serious pest species, such as the European and Asian corn borers, *Ostrinia* spp. and *Loxostege* spp. [13–16]. The North American fauna includes 157 species placed in 31 genera, 70% known from the western half of the continent [17–21]. Although the greatest number of species and genera occur in the temperate zone, this subfamily occurs on all continents except Antarctica. Interestingly, many genera are shared between the New and Old World tropics, some showing evidence of multiple interchanges. The possibility of a past faunal link via the north is indicated by the fact that many mainly tropical genera extend into subtropical and even temperate regions of North America and Asia [22].

Pyraustines are rather uniform in appearance as they possess triangular forewings, slender bodies, and are predominantly straw-yellow, brown or red, although some species are strikingly colored and a few are mimetic. They are usually easy to separate from other crambids including the Spilomelinae, a subfamily which is believed to be polyphyletic [23]. However, it is extremely difficult to identify pyraustines to a species level because of their subtle variation in wing colouration patterns. Therefore, the moths have been the subject of repeated taxonomic revisions [23–25].

This study represents an important step towards the development of a comprehensive DNA barcode library for North American Pyraustinae. It assembles 1589 sequences from 137 species in 25 genera, providing coverage for 87% of the fauna. We examine the correspondence between morphological traits and genetic divergence, and ask if barcodes can reveal species overlooked by past taxonomic work. Our results establish that DNA barcodes are effective for both identifying North American Pyraustinae and revealing overlooked species.

## Materials and Methods

### Sampling

**Fig 1:**
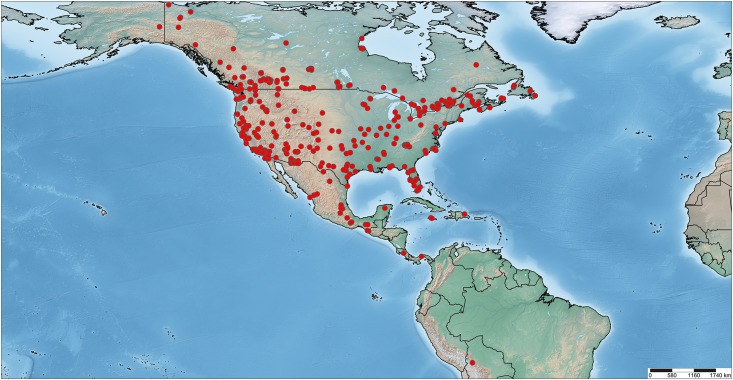
Distribution map of specimens of Pyraustinae sampled in this study with geocoordinates (n = 1461).

A total of 1648 specimens representing 142 known species and 10 provisional species in 27 genera were sampled from localities across North America (Fig 1). These specimens derived from 17 collections and most had been identified to a species-level by expert curators. When possible, several individuals of each species were analysed. Most specimens were from Canada, the United States, and Mexico, but a few specimens from Central America, the Caribbean, and South America for species whose distributions extend into the Neotropical Region were also included. We examined 524 specimens from the CNC as it holds a particularly strong collection of North American Pyraustinae, including 30 types determined by specialists such as EG Munroe, B Landry and A Mutuura.

### Morphology

Genital characters are predominantly used for discriminating species for pyralid moth and afford better diagnostic value than external wing patterns or colouration. More than 200 specimens were dissected to examine cases where there was a conflict between morphology-based identications and the DNA barcode results. Protocols for the preparation and photography of genitalic slides followed [26] while genital terminology followed [17, 27, 28]. Morphological analyses were performed following [20] in all difficult cases.

### DNA extraction, COI amplification and sequencing

Genomic DNA was extracted from a single leg from each dried specimen using a standard glass fibre protocol [29]. PCR amplification and DNA sequencing were performed at the Canadian Centre for DNA Barcoding following standard high-throughput protocols (http://ccdb.ca/resources.php). The primers LepF1 and LepR1 were first employed to amplify a full-length (658 bp) barcode region of the mitochondrial COI gene [9]. In cases where no product was recovered, failure tracking was carried out using multiple primer sets designed to recover shorter amplicons (164 bp, 295 bp, 307 bp, 407 bp) [30]. Each PCR reaction had a total volume of 12.5 μL, and contained 2 μL of DNA template, 6.25 μL of 10% D-(+)-trehalose dihydrate (Fluka Analytical), 2 μL of Hyclone ultrapure water (Thermo Scientific), 1.25 μL of 106 PlatinumTaq buffer (Invitrogen), 0.625 μL of 50 mM MgCl_2_ (Invitrogen), 0.125 μL of each primer, 0.0625 μL of 10 mM dNTP (KAPA Biosystems), and 0.060 μL of 5U/μL PlatinumTaq DNA Polymerase (Invitrogen). The thermocycling profile was 94°C for 1 min, 5 cycles at 94°C for 40 s, 45°C for 40 s, 72°C for 1 min, followed by 35 cycles 94°C for 40 s, 51°C for 40 s, 72°C for 1 min, with a final extension at 72°C for 5 min. The thermal cycle for shorter fragments was 94°C for 2 min, 5 cycles at 94°C for 40 s, 46°C for 1 min, 72°C for 30 s, followed by 35 cycles 94°C for 40 s, 53°C for 1 min, 72°C for 30 s, with a final extension at 72°C for 30 s [31]. PCR products were visualized on 2% agarose E-Gel 96 pre-cast gels (Invitrogen) and bidirectionally sequenced. Cycle sequencing employed a modified BigDye v3.1 Terminator (Applied Biosystems) protocol [32]. The thermocycling profile was 96°C for 1 min, followed by 35 cycles 96°C for 10 s, 55°C for 5 s, 60°C for 2.5 min, with a final extension at 60°C for 5 min. Sequencing was performed on an ABI 3730xl DNA Analyser (Applied Biosystems). Sequences were edited and assembled using CodonCode v. 3.0.1 (CodonCode Corporation).

### Sequence analysis

Sequences were aligned using CLUSTAL X and genetic distances within and between species were calculated in MEGA 6.0 employing the Kimura 2-parameter (K2P) model [33, 34]. MEGA 6.0 was also used to produce a Neighbor-Joining tree and for bootstrap analysis (1000 replicates). Complete specimen metadata including images, GPS coordinates, voucher depositories, sequences, trace files, and GenBank accession numbers are available in the Barcode of Life Data systems (BOLD, http://www.boldsystems.org) in the public dataset ‘DS-ZYPAN, Pyraustinae of North America’ which is available at the following dx.doi.org/10.5883/DS-ZYPAN.

The performance of two commonly used methods for OTU designation, Automatic Barcode Gap Discovery (ABGD, http://wwwabi.snv.jussieu.fr/public/abgd/abgdweb.html) [35] and the Barcode Index Number (BIN) system [36], were compared. ABGD analyses were performed online with two values of relative gap width (X = 1.0, 1.5) and three distance metrics (Jukes-Cantor, K2P, p-distance). Defaults were employed for all other parameter values, *P* (prior intraspecific divergence) ranged from 0.001 to 0.1 while Steps was set to 10, and Nb bins (for distance distribution) was set to 20. The four categories of BIN correspondence (MATCH, MERGE, SPLIT, MIXTURE) with known species are defined in [36].

## Results

As 1589 COI sequences were obtained from the 1648 specimens, sequencing success was high (96.4%). Most (86.8%) of the sequences ranged between 503–658 bp (mean 643 bp), but 6.2% were between 201–499 bp (mean 341 bp) and 7.0% were between 72–177 bp (mean 146 bp). Thirty of these sequences, ranging in length from 130–658 bp, were recovered from the type specimens of 14 species that ranged in age from 50 to 150 years. On average, 10.8 specimens were analysed per species (range = 1–67). Eighty-one species (59.1%) had four or more barcodes; 29 species (21.2%) possessed two or three records and 27 species (19.7%) had one. In total, 137 of the 157 (87%) species attempted were successfully barcoded. Barcoding failed for 20 extremely rare species. Among the 27 genera sampled, only two, *Epicorsia* and *Munroeodes*, failed to deliver sequences. Subsequent analyses focused on the 1379 barcodes from 115 described species and 10 provisional taxa, which met the criteria (>500bp, <1% n) for BIN assignment on BOLD. All cases involving conflicts between initial morphology-based identifications and the barcode results were resolved by the genitalic analysis, which showed that the correct identifications were those indicated by the DNA barcode results.

### (a) Genetic distances and identification success

**Fig 2:**
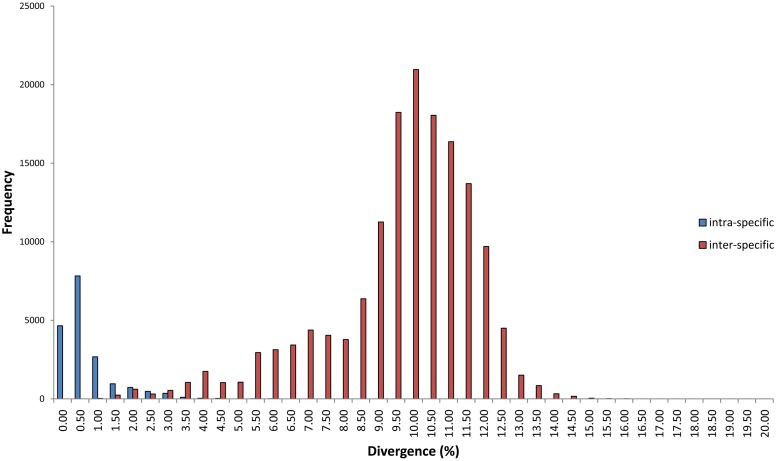
Frequency distribution of intraspecific and interspecific (congeneric) sequence divergence at COI in Pyraustinae involving17,832 intraspecific and 150,331 interspecific comparisons across 103 species. Divergences were calculated using the Kimura-2-parameter (K2P) model.

**Fig 3:**
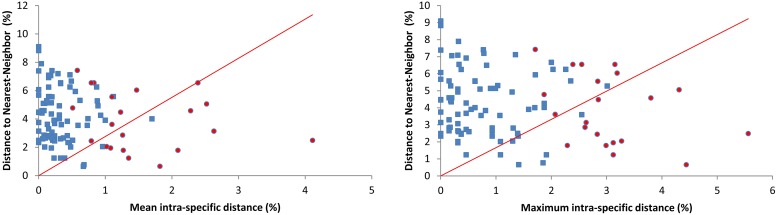
Mean intraspecific distance and maximum intraspecific distance (K2P) plotted against minimum distances to the nearest neighbor (NN) species for 103 species of Pyraustinae. Blue squares represent species assigned to a single BIN, while red circles are species placed in two or more BINs. Points above the diagonal line indicate species with a barcode gap.

Intraspecific genetic distances ranged from 0.00–5.56% (mean 0.50%), while interspecific divergences among congeners varied from 0.66–15.66% (mean 9.40%). Singletons were necessarily excluded from the analysis of intraspecific divergence. On average, the mean genetic distance between congeneric species was 18X higher than that within species (Fig 2, Table 1). The distance to the nearest neighbor (NN) species ranged from 0.66% to 9.10% (mean 4.25%). High intra-specific distance (>2%) was observed in 25 species while low inter-specific divergence (<2%) was found between 14 species (Fig 3) (assessment of distance thresholds in Lepidoptera based on [37]). Mean congeneric divergences varied from 2.64 to 9.95% with the highest value observed in the genus *Pyrausta*.

**Table 1:** Summary of Kimura 2-parameter genetic distances for the 103 species with two or more specimens, the 13 genera with two or more species.

Taxonomic Level	n	Taxa	Comparisons	Minimum Distance (%)	Mean Dist (%)	Maximum Dist (%)
Species	1357	103	17823	0	0.5	5.56
Genus	1259	13	150331	0.66	9.4	15.66

**Fig 4:**
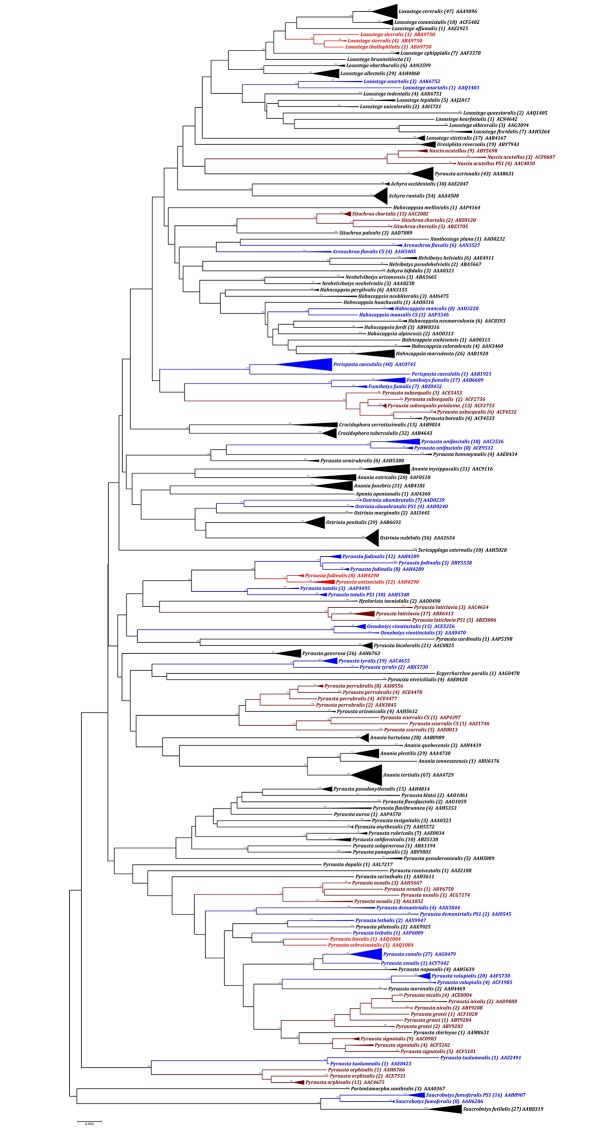
Neighbor-Joining tree (K2P) for 1379 COI sequences from Pyraustinae. The depth of each branch shows the divergence within each species. Numbers above branches indicate bootstrap values (>75 shown). Numerals in parenthesis following a species name indicate the number of individuals analyzed. Three letters followed by four numbers indicate the BIN. Taxa sharing a BIN are highlighted in light red, while paraphyletic and polyphyletic taxa are shown in blue and dark red respectively.

The specimens of most species were assigned to a single barcode cluster (Fig 4) with 99 of 125 species (79.2%) forming monophyletic clusters with high bootstrap support allowing their unambiguous identification. Three species pairs, *Loxostege thallophilalis*-*Loxostege sierralis*, *Pyrausta antisocialis*-*Pyrausta fodinalis*, and *Pyrausta linealis*-*Pyrausta ochreicostalis*, shared barcodes (4.8%), while 12 of the other 20 species were paraphyletic, and 8 were polyphyletic reflecting cases of deep intraspecific divergence. Interestingly, specimens of *P*. *fodinalis* were involved in both barcode sharing and deep divergence. When sequences in species with deep intraspecific divergence did not overlap with any other species, they were considered as delivering an identification, so the overall success rate of species identification was 95.2%.

### (b) Taxonomic performance of BIN and ABGD

All specimens in 99 of the species were assigned to distinct BINs that coincided with morphological species boundaries. The other 26 species were assigned to 56 BINs, reflecting cases of both deep intraspecific sequence divergence and barcode sharing, resulting in a total of 155 BINs. Although maximum intraspecific divergences in six species (*Anania funebris*-2.00%**,** *A*. *mysippusalis*-2.03%, *A*. *extricalis*-2.26%, *Crocidophora serratissimalis*-2.35%, *Hahncappsia marculenta*-2.55% and *Perispasta caeculalis*-3.01%) exceeded 2.0%, they were assigned to a single BIN, so BIN and species boundaries coincided even in these cases of high intraspecific divergence. By contrast, maximum intraspecific divergences in *Oenobotys vinotinctalis* and *Pyrausta volupialis* was less than 2.0% (1.71% and 1.87%, respectively), but they were each assigned to two BINs reflecting the fact that their component specimens fell into two distinct clusters. Interestingly, representatives of the clusters in each taxon occurred sympatrically.

**Fig 5:**
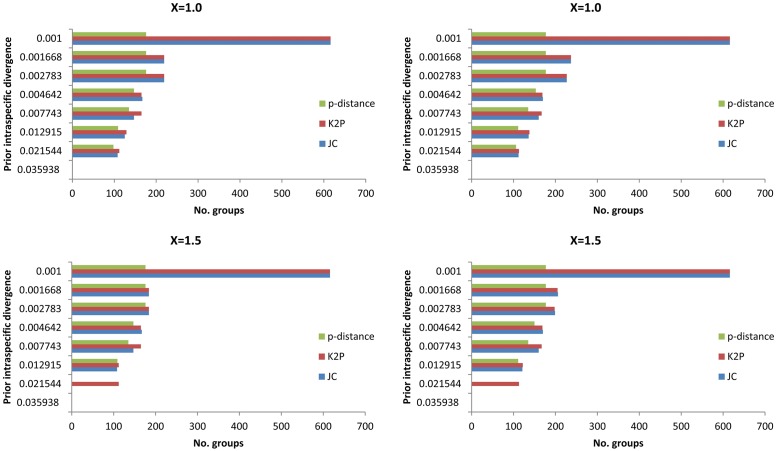
The automatic partition results by ABGD with three metrics and two X-values for 125 species of Pyraustinae. (a), (a') initial partitions; (b), (b') recursive partitions.

Two values of relative gap width (X = 1.0, 1.5) and three distance metrics (JC, K2P and p-distance) were used for both the initial and recursive partitions with ABGD. OTU counts resulting from varying values of prior intraspecific divergence (*P*) are shown in Fig 5 and Table 2. Initial partitions based on p-distances produced OTU counts ranging from 98 to 176 (X = 1.0) and from 109 to 176 (X = 1.5), while values ranged from 108 to 616 (X = 1.0, 1.5, respectively) with JC and from 112 to 616 (X = 1.0, 1.5, respectively) with K2P. Recursive partitions based on p-distances generated OTU counts ranging from 106 to 177 (X = 1.0) and from 111 to 177 (X = 1.5), whereas values with JC ranged from 112 to 616 (X = 1.0) and from 121 to 616 (X = 1.5) and with K2P from 113 to 616 (X = 1.0, 1.5, respectively). Both JC and K2P distance metrics produced more groups matching currently recognized species across a wider range of *P* values than did p-distance. The strongest correspondence was produced with *P* = 0.0129 under both JC (initial partitions: 125, 108, X = 1.0, 1.5, respectively; recursive partitions: 136, 121, X = 1.0, 1.5, respectively) and K2P (129, 112, X = 1.0, 1.5, respectively; recursive partitions: 138, 122, X = 1.0, 1.5, respectively) models.

**Table 2:** Results of partitions with two values of relative gap width and three distance metrics by ABGD.

Relative gap width	Prior intraspecific distance	Jukes-Cantor		K2P		p-distance	
		Initial Partition	Recursive partition	Initial Partition	Recursive partition	Initial Partition	Recursive partition
X = 1.0	0.035938	1	1	1	1	1	1
	0.021544	108	112	112	113	98	106
	0.012915	125	136	129	138	109	111
	0.007743	147	160	165	167	135	135
	0.004642	167	170	165	169	147	153
	0.002783	219	227	219	227	176	177
	0.001668	219	237	219	237	176	177
	0.001	616	616	616	616	176	177
X = 1.5	0.035938	0	0	1	1	0	0
	0.021544	1	1	112	113	1	1
	0.012915	108	121	112	122	109	111
	0.007743	147	160	165	167	135	135
	0.004642	167	170	165	169	147	150
	0.002783	184	199	184	198	176	177
	0.001668	184	206	184	205	176	177
	0.001	616	616	616	616	176	177

Number of groups in our data set includes 115 nominal species and 10 provisional species.

Our results revealed slight differences in the performance of BIN and ABGD (Table 3). The number of MATCH cases generated by BIN was intermediate, whereas it produced more SPLITS and fewer MERGES than ABGD. The number of MIXTURES was similar for both methods. In addition, the incidence of MATCHES for AGBD was affected by the values of relative gap width with the highest number of MATCHES (102) produced with the initial partition using K2P distance, X = 1.0 and *P* = 0.0129.

**Table 3:** The taxonomic performance of BIN and ABGD.

Method		Parameters	MATCH	SPLIT	MERGE	MIXTURE
BIN			99	20	5	1
ABGD	Initial partitions	JC, X = 1.0	100	7	16	2
		JC, X = 1.5	91	1	32	1
		K2P, X = 1.0	102	9	13	1
		K2P, X = 1.5	94	1	29	1
	Recursive partitions	JC, X = 1.0	98	11	14	2
		JC, X = 1.5	94	5	25	1
		K2P, X = 1.0	101	12	11	1
		K2P, X = 1.5	94	5	25	1

Number of groups in our data set includes 115 nominal species and 10 provisional taxa. Initial partitions and incursive partitions with *P* values (0.0129) and two values of relative gap width (1.5, 1.0) by ABGD are included. P-distance analyses were excluded from these statistical tests due to the strongly discordant results generated by this method.

### (c) Cases of barcode sharing or low divergence

Interspecific distances were lower than 2.0% for seven species pairs, but four of these pairs were assigned to distinct BINs (*Loxostege cereralis*-*L*. *commixtalis*, *Nascia acutellus-N*. *acutellus* PS1, *Pyrausta borealis*-*P*. *insequalis*, *Pyrausta grotei*-*P*. *nicalis*). By comparison, ABGD merged the members of these four species pairs in the initial partitions, with only *P*. *grotei*-*P*. *nicalis* discriminated by recursive partitions. As a result, BIN was better at discriminating these closely related species than AGBD. The other three pairs (*Loxostege sierralis-L*. *thallophilalis*, *Pyrausta antisocialis-P*. *fodinalis*, *Pyrausta linealis-P*. *ochreicostalis*) were placed in a single OTU by both BIN and ABGD, reflecting the fact that the species in each pair showed little or no divergence. Taxonomic notes on these taxa are provided in S1 Appendix.

### (d) Cases of DNA barcode paraphyly and polyphyly

Twenty of the 125 species (16%) displayed either a paraphyletic or polyphyletic topology in the NJ tree reflecting deep intraspecific variation. Taxonomic notes on these taxa are provided in S1 Appendix. Interestingly, 15 species (75% of these cases) involved *Pyrausta*, the most diverse genus in the subfamily with 58 known species placed in 18 species groups. Three of the polyphyletic species were *Pyrausta* (*P*. *insequalis*, *P*. *nexalis*, *P*. *perrubralis*) which were each assigned to four BINs. The other cases involved single species in five genera (*Fumibotys fumalis*, *Loxostege anartalis*, *Nascia acutellus*, *Oenobotys vinotinctalis*, and *Sitochroa chortalis*) which each divided into two or three BINs.

ABGD and BIN classified the lineages of these 20 para- and polyphyletic taxa in slightly different ways. Initial partitions with ABGD produced 12 MATCHES with a relative gap width (X = 1.5) under both JC and K2P models, and 8 MATCHES with a relative gap width (X = 1.0). Seven of these MATCHES (*F*. *fumalis*, *O*. *vinotinctalis*, *P*. *signatalis*, *P*. *tyralis*, *P*. *unifascialis*, *P*. *volupialis*, *S*. *chortalis*) were consistent with morphological identifications. *Loxostege anartalis* was the only SPLIT, while the lineages of four species (*N*. *acutellus*, *P*. *grotei*, *P*. *insequalis*, *P*. *nicalis*) were MERGED. OTU assignments were uncertain for the other eight species of *Pyrausta* (*P*. *laticlavia*, *P*. *zonalis*, *P*. *nexalis*, *P*. *orphisalis*, *P*. *perrubralis*, *P*. *scurralis* CS, *P*. *tuolumnalis*, *P*. *lethalis*) because outcomes varied with different parameter values of ABGD.

Genetic distances and sequence diversity values are summarized in Table 4. The maximum intraspecific genetic distances ranged from 1.71 to 5.56%, while the mean intraspecific distances varied from 0.51–4.11% and exceeded 2.00% in six taxa (*L*. *anartalis*, *P*. *grotei*, *P*. *lethalis*, *P*. *nexalis*, *P*. *scurralis* CS, *P*. *tuolumnalis*). The number of haplotypes per species ranged from 2 to 24. Nucleotide diversity (*Pi*) ranged from 0.00484–0.03976, while haplotype diversity (*Hd*) varied from 0.537–1.000. The highest nucleotide diversity (0.03976) was found in *P*. *lethalis*, reflecting deep lineage divergences among its allopatric populations. Four species (*L*. *anartalis*, *P*. *grotei*, *P*. *lethalis*, *P*. *scurralis* CS, *P*. *tuolumnalis*) had the highest haplotype diversity (1.00).

**Table 4:** The BINs, initial partitions by ABGD and genetic distance, sequence diversity of the paraphyletic and polyhyletic taxa in this study.

Taxon	No. of BINs	ABGD (JC, K2P, P = 0.0129)		Minimum distance (%)	Mean distance (%)	Maximum distance (%)	Nucleotide diversity (Pi)	Number of Haplotypes	Haplotype diversity (Hd)
		X = 1.0	X = 1.5						
*Fumibotys fumalis*	2	MATCH	MATCH	0	1.47	3.19	0.01521	19	0.975
*Loxostege anartalis*	2	SPLIT	SPLIT	0.77	2.28	3.8	0.02217	4	1.000
*Nascia acutellus*	2	MERGE	MERGE	0	1.08	3.12	0.01359	10	0.962
*Oenobotys vinotinctalis*	2	MATCH	MATCH	0	0.58	1.71	0.00570	6	0.562
*Pyrausta grotei*	3	MERGE	MERGE	0.31	2.09	2.99	0.02039	4	1.000
*Pyrausta insequalis*	3	MERGE	MERGE	0	1.35	3.12	0.01285	6	0.710
*Pyrausta laticlavia*	2	SPLIT/MIXTURE	MERGE	0	1.02	3.27	0.00987	11	0.895
*Pyrausta lethalis*	2	SPLIT	MIXTURE	1.71	4.11	5.56	0.03976	3	1.000
*Pyrausta nexalis*	4	SPLIT	MATCH	0	2.52	4.31	0.02307	7	0.964
*Pyrausta nicalis*	3	MERGE	MERGE	0	1.27	2.29	0.01378	5	0.786
*Pyrausta orphisalis*	3	SPLIT	MATCH	0	0.83	2.55	0.00719	6	0.617
*Pyrausta perrubralis*	4	SPLIT	MATCH	0	1.26	2.61	0.01166	6	0.817
*Pyrausta scurralis* CS	2	SPLIT	MATCH	2.63	2.63	2.63	0.02559	2	1.000
*Pyrausta signatalis*	3	MATCH	MATCH	0	1.1	2.07	0.01110	7	0.791
*Pyrausta tuolumnalis*	2	SPLIT	MATCH	2.39	2.39	2.39	0.02339	2	1.000
*Pyrausta tyralis*	2	MATCH	MATCH	0	0.79	3.15	0.00683	10	0.886
*Pyrausta unifascialis*	2	MATCH	MATCH	0	1.1	2.84	0.01337	6	0.605
*Pyrausta volupialis*	2	MATCH	MATCH	0	0.51	1.87	0.00484	7	0.537
*Pyrausta zonalis*	2	MATCH	MERGE	0	0.79	2.83	0.00744	24	0.950
*Sitochroa chortalis*	3	MATCH	MATCH	0	1.23	2.85	0.01315	9	0.827

## Discussion

Although valid identifications are critical for biodiversity monitoring, the very large number of closely related genera and sibling species within the Pyraustinae means that this subfamily presents a particular challenge. In fact, [17] indicated that careful analyses of intraspecific and interspecific differences are critical to confirm species identities and to reveal cryptic diversity within this subfamily. Because of the difficulty in morphology-based identifications, this group provides an excellent opportunity to test the efficacy of barcode-based species delimitation.

In the present study, we examined the utility of DNA barcoding for species identification in the Pyraustinae. Our results indicate that the barcode clusters were coincident with 79.2% of species boundaries as currently defined from morphology. However, identification success rose to 95.2% when cases of deep intraspecific variation were recognized as identifications, a success rate similar to those reported in prior studies on Lepidoptera [6–8, 38–41]. Earlier work has demonstrated that the analysis of more than 20 individuals per species improves the efficacy of DNA barcoding [42–45]. As only a quarter of the species (29 of 125) in our study reached this target, it is desirable to increase sample sizes for many species.

The performances of BIN and ABGD have been compared in several previous studies [35, 36, 46]. For example, [36] found that the BIN system generally outperformed ABGD. Our results indicate that the BIN system was better than ABGD in discriminating closely related species. As well, two values of relative gap width had substantial effects on the number of OTUs recognized by ABGD, meaning that it could not be used to predict species numbers. As noted in other studies, our results showed that ABGD tends to lump species by increasing the number of MERGES [36, 46, 47]. However, the relatively high incidence of SPLITS detected by BIN analysis suggests that there are many overlooked/cryptic species within the Pyraustinae. In addition, our analyses confirm the prior observation [35, 48] that recursive partitions in AGBD recognize more OTUs than primary ones, reflecting their superior capacity to deal with variation in sample sizes of the species under analysis. However, the initial partitions generated more constrained counts, which showed the closest match to currently recognized species with *P* = 0.01 under both JC and K2P models.

Deep sequence divergences were detected in 16% (20/125) of the species of Pyraustinae that we examined, a higher value than noted in previous studies [38–41]. Most of these cases involved *Pyrausta*, one of most difficult genera in North American Lepidoptera. Because of its high diversity and lack of conspicuous diagnostic morphological characters, it is likely that there are many overlooked species in this genus. ABGD generated slightly different results than the BIN system, but eight SPLITS (*L*. *anartalis*, *P*. *laticlavia*, *P*. *lethalis*, *P*. *nexalis*, *P*. *orphisalis*, *P*. *perrubralis*, *P*. *scurralis* CS, *P*. *tuolumnalis*) were recognized by both BIN and ABGD. These cases are particularly likely to represent cryptic taxa, and preliminary examination indicates previously overlooked morphological differences between lineages in these taxa. While it is possible that some cases reflect intraspecific variation that arose in allopatry [7, 40, 41], careful morphological studies of male and female genital structures will likely raise the species count for North America [20].

We found seven species pairs which either shared barcodes or had low divergence. Although barcode sharing can arise through mitochondrial introgression or incomplete lineage sorting, it can also reflect unrecognized synonymy [7, 39, 40]. Four of these pairs (eg. *Loxostege cereralis*-*L*. *commixtalis*, *Nascia acutellus-N*. *acutellus* PS1, *Pyrausta borealis*-*P*. *insequalis*, *Pyrausta grotei*-*P*. *nicalis*) showed enough sequence divergence to be assigned to different BINs, but were placed in single OTUs by ABGD. The other three species pairs (*Loxostege sierralis-L*. *thallophilalis*, *Pyrausta antisocialis-P*. *fodinalis*, *Pyrausta linealis-P*. *ochreicostalis*) had similar barcodes so they may be very young species pairs or cases of synonymy. Two species (*Pyrausta fodinalis*, *Pyrausta lethalis*) were classified as MIXTURES by BIN and ABGD respectively, suggesting that historical hybridization events may have led to rare bidirectional introgression.

Short segments of the barcode region are often effective for species identification and can ordinarily be recovered from museum samples [31, 49, 50] via both Sanger or next-generation sequencing and have helped to resolve longstanding taxonomic uncertainty [20, 51–56]. In this study, we excluded 210 mini-barcodes from analysis because they were not long enough to obtain a BIN assignment. However, 90% of the 30 sequences recovered from type material of 14 species matched sequences from recently collected conspecifics, indicating the utility of these short sequences to confirm current name use. The four exceptions involved sequences that were too short to allow species discrimination.

The DNA barcode reference library for Lepidoptera in BOLD (http://www.boldsystems.org) [57] now includes records for more than 98,000 named species. Work is underway to gather barcodes for all Lepidoptera species from North America and records are now available for more than 8500 of the 13,000+ species known from Canada and the United States, including 550 species of Crambidae (http://www.lepbarcoding.org). The present study has compiled a comprehensive DNA barcode reference library for the Pyraustinae from North America, enabling anyone with access to sequencing resources to identify unknown specimens in this group and aiding assessments of their diversity.

## Supporting Information

S1 Appendix Taxonomic notes on cases of barcode sharing, paraphyletic and polyphyletic species. (PDF)

## Abbreviations

AMNH: American Museum of Natural History
BIO: Biodiversity Institute of Ontario
CNC: Canadian National Collection of Insects, Arachnids and Nematodes
COC: College of Charleston
MEM: Mississippi Entomological Museum
NCBI: Mined from GenBank
RBCM: Royal British Columbia Museum
RCBS: Research Collection of Brian Scholtens
RCDH: Research Collection of Daniel Handfield
RCJBS: Research Collection of J. Bo Sullivan
RCJW: Research Collection of Jeremy deWaard
ROM: Royal Ontario Museum
STRI: Smithsonian Tropical Research Institute
TAMU: Texas A&M University, College Station
UOD: University of Delaware
UOG: University of Guelph
UOM: University of Maryland
WSDA: Washington State Department of Agriculture
