# Supplementary material for: A DNA Barcode Library for North American Pyraustinae (Lepidoptera: Pyraloidea: Crambidae)
Source: PLoS One. 2016 Oct 13;11(10):e0161449. doi: 10.1371/journal.pone.0161449 (PMC5063472; doi:10.1371/journal.pone.0161449)
Supplement: S1 Appendix — (PDF) [file pone.0161449.s001.pdf]

S1 Appendix: Taxonomic notes on cases of barcode sharing, paraphyletic and polyphyletic species.

- **Cases of barcode sharing**

1. ***Loxostege sierralis* Munroe, 1976** Type locality: USA, California, 7 mi WSW of Lee Vining, 9,600 feet. [CNC]

   ***Loxostege thallophilalis* (Hulst, 1886)** Type locality: USA, California; Montana. [AMNH]

Both species are closely similar to *Loxostege brunneitincta* Munroe, 1976 (type locality: USA, California, Mount Shasta, Timberline. [CNC]), *Loxostege cereralis* (Zeller, 1872) (type locality: Texas. [BMNH]), *Loxostege commixtalis* (Walker, 1866) (type locality: Hudson's Bay, Albany River, St. Martin's Falls. [BMNH]) and *Loxostege offumalis* (Hulst, 1886) (type locality: USA, California, San Diego. [AMNH]). Munroe (1976) described *Loxostege sierralis* as a new species and recognized four subspecies, represented by *L. s. internationalis* Munroe, 1976 (type locality: Canada, British Columbia, Blackwall, Manning Park, 6,000 feet. [CNC]), *L. s. sanpetealis* Munroe, 1976 (type locality: USA, Utah, Sanpete County, 17 mi E of Mayfield. [AMNH]), *L. s. sierralis* Munroe, 1976 (type locality: type locality: USA, California, 7 mi WSW of Lee Vining, 9,600 feet. [CNC]) and *L. s. tularealis* Munroe, 1976 (type locality: USA, California, Tulare, Big Meadows. [LACM]). He also recognized *Noctuelia flavifimbrialis* Warren, 1892 (type locality: USA, California. [BMNH]) as a synonymy of *Loxostege thallophilalis*. In our analyses, *Loxostege sierralis*, a montane species of the western Cordillera, sampled from California, Montana and British Columbia shared barcodes with *Loxostege thrallophilalis* from Washington. The genetic distance between species varied from 0.77 to 1.71% (mean 1.02%). Mitochondrial introgression is a possible explanation for the shared barcodes between these two taxa. Further investigation by using multi-loci (e.g., nuclear genes) would help to resolve the problem of these two sister species that are likely broadly sympatric and may hybridize frequently.

Nomenclatural note: The spelling "*thrallophilalis*" in the original description by

Hulst (1886, p. 154) was an inadvertent error corrected by Hulst himself at the end of the same publication (p. 168). Because this is clear evidence of an error in the original publication, *thallophilalis* must be accepted as the correct original spelling (ICZN 1999, Art. 32.5.1.1). Barnes & McDunnough (1917) as well as Munroe (1976, 1983) and Scholtens & Solis (2015) used the correct spelling *thallophilalis*, but McDunnough in the 1939 *Check List* incorrectly listed it as “*thrallophilalis*”.

2. ***Pyrausta antisocialis* Munroe, 1976** Type locality: USA, New Mexico, McKinley County, Zuñi Mountains, McGaffey, 7,500 feet. [CNC]

***Pyrausta fodinalis* (Lederer, 1863)** Type locality: USA, California. [BMNH]

Pyrausta antisocialis and *Pyrausta fodinalis* are closely related with another species *Pyrausta socialis* (Grote), 1877 (type locality: probably near London, Ontario, Canada. [BMNH]), these species were placed in *fodinalis* group. *Pyrausta antisocialis* is recorded only at high elevations in New Mexico and Arizona, whereas both *Pyrausta socialis* and *Pyrausta fodinalis* are transcontinental in range but seemingly absent from the Southwest. Munroe (1951; 1976) indicated that male genitalia of *Pyrausta fodinalis* can be easily distinguished from those of two allied species. Munroe (1976) recognized three subspecies of *Pyrausta fodinalis* and two subspecies of *Pyrausta socialis*, namely *P. f. fodinalis* (Lederer), 1863 (type locality: USA, California. [BMNH]), *P. f. monticola* Munroe, 1976 (type locality: USA, California, Siskiyou Co., Mt. Shasta. [CNC]), *P. f. septentrionicola* Munroe, 1976 (type locality: Alberta, Scandia. [CNC]), *P. s. socialis* (Grote), 1877 (type locality: Canada. [BMNH]) and *P. s. perpallidalis* Munroe, 1976 (type locality: USA, Washington, Yakima Co., Kusshi Canyon. [CNC]). In our analyses, three clusters were displayed for *Pyrausta antisocialis* and *Pyrausta fodinalis*. Interestingly, eight records of *P. f. monticola* sampled from Utah, Oregon, California and Alberta shared barcode with *Pyrausta antisocialis* from Western North America including three records from New Mexico and Arizona as same collecting data as types. The genetic distance between species varied from 0.66 to 1.88% (mean 1.30%). However, other records of *Pyrausta fodinalis* from Alberta formed another two distinct clusters. Although this represent an uncommon case, we believe that these never lead to misidentifications because *P.*

antisocialis can be easily distinguished from those of *P. fodinalis* in having the uncus more slender and provided with a lateral point on either side at the tip in male genitalia. Therefore, we suspect that very recent speciation or extensive introgressive hybridization may have occurred leading to this rare case of barcode sharing. In addition, the status of three subspecies of *Pyrausta fodinalis* should be reconsidered according to our analysis based on our morphological and molecular data.

3. ***Pyrausta linealis* (Fernald, 1894)** Type locality: USA, California, Argus Mts. [USNM]

   ***Pyrausta ochreicostalis* Barnes & McDunnough, 1918** Type locality: USA, California, Riverside Co., Palm Springs. [USNM]

Pyrausta linealis with markings and genitalia is most similar as in *Pyrausta ochreicostalis* but darker and without the prominent fulvous costal area of that species. The former occurs from the interior of Washington to the Mojave Desert in California and the latter species ranges from Apple Valley to Borrego, California, and east to Valley of Fire in Southern Nevada. Munroe (1976) retained *Pyrausta linealis* and *Pyrausta ochreicostalis* as two valid species even though intermediates between the two species are common in nature. In our analyses, *Pyrausta linealis* and *Pyrausta ochreicostalis* shared identical barcodes and the genetic divergence ranged from 1.21 to1.86% (mean 1.48%) between them. Our finding implied that the fact some individuals with intermediate patterns are often particularly difficult to identify. Further investigations are desirable for delimiting those two species based on examination of the types, and the possibility of synonymy should also be considered.

- **Cases of polyphyly and paraphyly**

1. ***Fumibotys fumalis* (Guenée, 1854).** Type locality: USA, Georgia. [USNM]

This species is an economic pest feeding on rhizomes of peppermint and other mints and occurs from Nova Scotia to British Columbia and Washington and south to Florida, eastern Texas and Utah. Munroe (1976) treated *Fumibotys fumalis* a new combination and recognized two synonyms, *Scopula orasusalis* Walker, 1859 (type locality: Canada, Nova Sotia. [BMNH]), and *Botis badipennis* Grote, 1873 (type

locality: USA, Maine. [BMNH]). Interestingly, two distinct clusters were displayed and the genetic distance was higher than 2.0% between them. One sampled across the United States and Canada should be *Fumibotys fumalis*, whereas another should be a putative species found in New Brunswick and Ontario, Canada. We dissected two specimens for *Fumibotys fumalis* but none of the putative species. More investigation is needed.

2. ***Loxostege anartalis* (Grote, 1877)** Type locality: USA, California, Soda springs. [BMNH]

This species is much as *Loxostege immerens* (Harvey, 1875) (type locality: USA, California. [USNM]) and ranges from eastern Quebec to Alaska, south to the southern Prairie Provinces and in the mountains to Utah and northern California. Munroe (1976) concluded that five more or less well-marked subspecies be included within *Loxostege anartalis*, namely *L. a. albertalis* Barnes & McDunnough, 1918 (type locality: Alberta, Gleichen. [USNM]), *L. a. anartalis* (Grote), 1877 (type locality: USA, California, Soda springs. [BMNH]), *L. a. lulualis* (Hulst), 1886 (type locality: USA, California. Canada, Quebec, Anticosti Island. [AMNH]), *L. a. rainierensis* Munroe, 1976 (type locality: Washington, Mt. Rainier. [CNC]), *L. a. saxicolalis* Barnes & McDunnough, 1918 (type locality: Utah, Stockton. [USNM]). Barnes & McDunnough (1918) treated *albertalis* as specifically distinct from the nominal subspecies and proposed the name *albertalis* for the species. In our analyses, two distinct clusters were displayed for *Loxostege anartalis*, one contains two records from the Northwest Territories, Canada (identified as *L. a. albertalis*), and another represents all remaining subspecies including records from the Northwest. We compared the male genitalia of *L. a. albertalis* with other subspecies, but it is nearly indistinguishable except for having subtle differences in aedeagus. It will be necessary to study the types of *albertalis* subspecies and to conduct a morphometric study of genital structures to confirm diagnostic features and decide whether some may represent full species.

3. ***Nascia acutellus* (Walker, 1866)** Type locality: Canada, Ontario. [BMNH]

Nascia acutellus and its sibling species *Nascia cilialis* (Hübner), 1796 (type

locality: Oesterreich) are Holarctic species. As mentioned in Munroe (1976), the former is confined to the eastern part of North America, whereas the latter ranges from Europe to Japan with some geographical variation. Munroe (1976) treated *Nascia acutellus* as a new combination and recognized a synonym, *Botis venalis* (Grote, 1878) (type locality: [Evans Center, near] Buffalo, New York. [BMNH]). In our analyses, *Nascia acutellus* had three distinct clusters and the genetic distance was higher than 2.0% between each other: this is congruent with our morphological study of genital structures which revealed three different morphospecies. We suspect that two cryptic species have been overlooked and are widespread in eastern North America. Whether one of them matches the synonym *venalis* will require examination of the type.

4. ***Oenobotys vinotinctalis* (Hampson, 1895)** Type locality: West Indies, Grenada, Balthazar. [BMNH]

This species has been recorded from North Carolina to Florida, west to Texas, and southward through West Indies and Mexico to Central and South America. Munroe (1976) treated it as a new combination and erected the genus *Oenobotys*. Munroe & Blanchard (1976) described *Oenobotys texanalis* (type locality: USA, Texas, Jeff Davis Co., Fort Davis. [CNC]) as a new species, which is a larger moth with distal half of fringe contrastingly white and only recorded from Texas. Two distinct clusters were displayed of *Oenobotys vinotinctalis*. However, the genetic distance was lower than 2.0%. One includes three records from Florida and another includes a few records from Florida, Mississippi and North Carolina. We suspect that three specimens from Florida are most likely *Oenobotys texanalis* based on the characteristic feature of the fringe, however further analysis is needed to confirm if they are correctly identified.

5. ***Pyrausta grotei* (Munroe, 1976)** Type locality: USA, Colorado. [BMNH]

This mainly montane species occurs from eastern Washington and Wyoming to California, ranging up to 10,000 feet in Arizona. Munroe (1976) considered *Botis augustalis* Grote, 1881(type locality: USA, Colorado. [BMNH]) as a synonym of *Pyrausta grotei*. Three distinct clusters were displayed and the genetic distance was

higher than 2.0% between each other. One includes two records from California, another two clusters includes one from California and another from Texas and Arizona. Our results suggest that *augustalis* may be a distinct, valid species, but more detailed study is needed.

6. ***Pyrausta insequalis* (Guenée, 1854)** Type locality: Amérique septentrionale.

This species is likely confused with *Pyrausta tatalis* (Grote, 1877) (Type locality: USA, Texas, Belfrage. [BMNH]) and *Pyrausta semirubralis* (Packard, 1873) (type locality: USA, California) and ranges from Newfoundland and Labrador to Alaska, south to northern Florida, California, and the mountains of Arizona, New Mexico and Mexico. Munroe (1976) indicated that *Herbula insequalis* Guenée, 1854 was an unnecessary replacement name for *H. subsequalis* Guenée, 1854, because there is no homonymy with *Botys subsequalis* Herrich-Schäffer, 1871. He recognized four subspecies, represented by *P. s. borealis* Packard, 1867 (type locality: Square Island, Labrador), *P. s. petaluma* Munroe, 1976 (type locality: Petaluma, Sonoma Co., California. [CNC]), *P. s. plagalis* Haimbach, 1908 (type locality: Miller's Canyon, Huachuca Mts., Arizona. [USNM]) and *P. s. subsequalis* (Guenée, 1854) (type locality: was not clearly indicated, North America. [USNM]). He also indicated that the European *Pyrausta cespitalis* ([Denis & Schiffermüller], 1775) (type locality: [Austria] Wienergegend) and its representatives in temperate Asia are probably only subspecifically different. Munroe (1983) followed Munroe (1976) and treated *insequalis* as a synonym of *subsequalis*. But Munroe *et al.* (1995) used *insequalis* in Atlas of Neotropical Lepidoptera and considered *subsequalis* as a synonym of *insequalis*. He also recognized *P. i. fascetalis* (Berg, 1874) as a new combination and treated *cespitalis* as a synonym of subspecies *fascetalis*. Poole (1996) listed *borealis* as a species. Scholtens & Solis (2015) followed Munroe *et al.* (1995) and Poole (1996) and listed three subspecies of *Pyrausta insequalis*, namely *P. i. plagalis*, *P. i. petaluma* and *P. i. fascetalis*. In our analyses, four distinct clusters of *Pyrausta insequalis* and one cluster of *Pyrausta borealis* (Alberta, British Columbia and Quebec, Canada) were displayed. Interestingly, the genetic distance was lower than among three clusters of *Pyrausta insequalis*. One was identified as *P. i. plagalis* and another *P. i.*

petaluma including specimens from Western of North America. The third one was an undetermined subspecies from Mexico. However, *P. i. plagalis* mostly sampled from eastern North America, including Quebec, Oklahoma and Mississippi (with one exception from Arizona) diverged from the other three clusters of *Pyrausta insequalis* but clustered together with *Pyrausta borealis*. The genetic distance was lower than 2.0% between eastern *P. i. plagalis* and *Pyrausta borealis* but higher than 2.0% between eastern *P. i. plagalis* and Western *P. i. plagalis* as well between eastern *P. i. plagalis* and *P. i. petaluma*. By contrast, the genetic distance was less than 2.0% between *Pyrausta borealis* and western *P. i. plagalis* as well between *Pyrausta borealis* and *P. i. petaluma*. Our findings suggest that the genetic diversity of *P. insequalis* is most likely related to geographical variation and that historical introgression may have occurred between eastern *P. i. plagalis* and *Pyrausta borealis*, but more investigations are desirable.

7. ***Pyrausta laticlavia* (Grote & Robinson, 1867)** Type locality: USA, Pennsylvania. [AMNH]

This species somewhat resembles *Pyrausta tyralis* (Guenée) (type locality: Cuba), 1854 and ranges from New Jersey to southern Florida, Missouri, southern Texas, Oklahoma and southern California. Munroe (1976) recognized a variable series in *Pyrausta laticlavia* and treated *Botys cinerosa* Grote & Robinson, 1867 (type locality: USA, Pennsylvania) as a synonym, which was considered as a transitions form with both yellow and pink areas infuscated and having markings cinereous not yellow by comparing with *laticlavia*. Powell & Opler (2009) pointed out that *Pyrausta laticlavia* has greater seasonal variation than *Pyrausta* spp., summer moths having bright pink forewings with fuzzy yellowish bands whereas those flying in fall and early spring are much darker, almost uniform dark grey brown in the most extreme individuals. In a long, reared series from southeastern Michigan (CNC) with all moths having emerged in late August and from which we barcoded three specimens (in BIN BOLD:ABX6413), we observed an obvious sexual dichromatism without intermediates, males having pale areas of the wings yellow whereas females have them grey. In our analysis, three distinct clusters were displayed and the genetic

distance was higher than 2.0% between each other. Our results suggest that two cryptic species may have been overlooked, but the variation in this case deserves further morphological scrutiny.

8. ***Pyrausta lethalis* (Grote, 1881)** Type locality: USA, California, Havilah. [BMNH]

This species is most likely to be confused with *Pyrausta corinthalis* Barnes & McDunnough, 1914 (type locality: USA, Arizona, Palmerlee. [USNM]) and ranges from Los Angeles, California, through the Mojave Desert to southern Nevada, and east through southern Arizona to Texas. Munroe (1976) stated that there is substantial variation in different series of *Pyrausta lethalis*. Texas specimens are small and fulvous while those from Arizona tend to be reddish. In California each sample seems to have its own characteristics. In our analyses, two records from Arizona and Texas formed a cluster whereas other records from California formed another distinct cluster. Our morphological study showed that differential diagnosis in genital structure is subtle based on the females from Arizona and California, but it is difficult to separate them with the limited material at hand. Further study is desirable.

9. ***Pyrausta nexalis* (Hulst, 1886)** Type locality: USA, California, Sierra Nevada Mountains. [AMNH]

Pyrausta nexalis is similar to *P. zonalis* Barnes & McDunnough, 1918 (type locality: USA, California, Riverside Co., Palm Springs. [USNM]) and ranges from Washington to Montana, south to Texas and southern California. Fernald (in Dyar, [1903]) transferred *Botis nexalis* Hulst, 1886 to *Autocosmia* and sank *Autocosmia concinna* Warren, 1892 (type locality: "N. W. America", Walsingham. [BMNH]) as a synonym, but Barnes and McDunnough (1917) listed the two as separate species of *Autocosmia* in their checklist. Munroe (1976) transferred two to *Pyrausta* and treated *concinna* as a synonym. He also indicated that *Pyrausta nexalis* varies in size and in the amount of fulvous suffusion on the forewing. Specimens from the California Sierra tend to be large, and those from the eastern part of the range have more fulvous than those from the West. In our analysis, four distinct clusters were displayed for *Pyrausta nexalis*. However, the genetic distance was lower than 2.0% between two

clusters, and examination of their male genitalia indicated that they were all *Pyrausta nexalis*. One cluster includes three records from Washington and Arizona and another includes three samples from Texas and Arizona. Three records from California and Texas and one record from California formed two additional distinct clusters. Our results suggest the presence of overlooked cryptic species. But more material and investigation are needed to assess the significance of the morphological variation of *Pyrausta nexalis* related to genetic divergence.

10. ***Pyrausta nicalis* (Grote, 1878)** Type locality: USA, California, Sierra Nevada. [BMNH]

This species is similar in general aspect to *Pyrausta grotei* (Munroe, 1976) (Type locality: USA, Colorado. [BMNH]), and both were placed in the *nexalis* species group. *Pyrausta nicalis* has been recorded from Quebec, across Canada to British Columbia, and south to Colorado, Utah, Nevada and California. Munroe (1976) recognized two synonyms of *Pyrausta nicalis*, representing by *Botis uxorculalis* Hulst, 1886 (type locality: Sierra Nevada Mountains California, USA. [AMNH]) and *Syllythria subnicalis* Warren, 1892 (type locality: California USA. [BMNH]). He also pointed out that all three names were based on material from the same region and that there was some morphological variation. Interestingly, our analyses revealed three distinct clusters. One should be the real *Pyrausta nicalis* based on our morphological study, including two records sampled from Utah and Nevada, United States. Another two clusters from Western Canada may represent two distinct species that have been overlooked. In addition, the taxonomic status of *uxorculalis* and *subnicalis* should be re-examined to assess whether they may deserve valid species status.

11. ***Pyrausta orphisalis* Walker, 1859** Type locality: USA, Ontario, Albany R., St. Martin's Falls. [BMNH]

This species is often confused with *Pyrausta tuolumnalis* Barnes & McDunnough, 1918 (type locality: USA, California, Tuolumne Meadows. [USNM]) and *Pyrausta generosa* (Grote & Robinson), 1867 (type locality: USA, Pennsylvania [AMNH]). *Pyrausta orphisalis* is known from Newfoundland across southern Canada to British Columbia, south to northern Florida, New Mexico and California. Munroe

(1976) indicated that it is even more closely related to the European *Pyrausta aurata* (Scopoli), 1763. He also considered *Pyrausta ochosalis* Dyar, 1903 (type locality: New York. [USNM]) to be a synonym. In our analyses, three distinct clusters were displayed each separated by more than 2.0% from the nearest neighbor, implying the presence of overlooked cryptic taxa with similar morphology. The first one includes three records from Ontario, Canada; the second includes single record from Michigan, United States; the third distinct cluster includes the remaining records from across the United States and Canada. We dissected three female specimens for each of clusters, as none of specimens are male. Further study based on the male genitalic characters is needed.

12. ***Pyrausta perrubralis*** **(Packard, 1873)** Type locality: USA, California.

This species is closely similar to *Pyrausta scurralis* (Hulst), 1886 (type locality: USA, Arizona. [AMNH]) and ranges from Vancouver Island to southern California, as well as in the Sierra Nevada. Munroe (1951; 1976) recognized three subspecies: *P. p. shastanalis* Munroe, 1976 (type locality: USA, California, Mt. Shasta. [CNC]); *P. p. saanichalis* Munroe, 1951 (type locality: Canada, British Columbia, Duncan. [CNC]) ; and *P. p. perrubralis* (Packard), 1873 (type locality: USA, California). In our analysis, four distinct clusters of *Pyrausta perrubralis* were displayed. However, one comprising four records including the allotype of *P. p. shastanalis* formed a distinct cluster more than 2.0% distant from the other three clusters. By contrast, the genetic distance was lower than 2.0% among another three clusters. Our findings suggest that *P. p. shastanalis* should be elevated to full species level. We retain its subspecies rank at present pending a detailed investigation of genital morphology.

13. ***Pyrausta scurralis*** **(Hulst, 1886)** Type locality: USA, Arizona. [AMNH]

This species is similar and formerly was considered to be an inland race of *Pyrausta perrubralis* (Packard, 1873) (type locality: USA, California) ranging from Manitoba and Saskatchewan to Arizona, New Mexico and the state of Durango, Mexico (Munroe, 1976; Powell & Opler, 2009). Munroe (1976) raised it to be a full species and recognized two subspecies, *P. s. awemealis* Munroe, 1976 (type locality: Canada, Manitoba, Aweme. [CNC]) and *P. s. scurralis* (Hulst, 1886) (type locality:

USA, California). He also treated *Pyrausta postrubralis* Hampson, 1899 (type locality: USA, Arizona. [BMNH]) as a new synonymy with *Pyrausta scurralis*. Three distinct clusters of *Pyrausta scurralis* were displayed. Each of two distinct clusters includes only a single record sampled from Mexico and Arizona respectively, the latter identified as *P. s. scurralis*. Other records from USA and Canada formed another cluster which matched *P. s. awemealis*. According to our morphological analysis, we suspect that a cryptic species has been overlooked and that *P. s. awemealis* should be raised to species level. More material and further study are needed.

14. ***Pyrausta signatalis* (Walker, 1866)** Type locality: North America. [BMNH]

This species is closest to *Pyrausta pythialis* Barnes & McDunnough, 1918 (type locality: USA, Manitoba, Cartwright. [USNM]) and *Pyrausta inveterascalis* Barnes & McDunnough, 1918 (type locality: USA, Pennsylvania, New Brighton. [USNM]) and ranges from Ottawa, Ontario, to the Carolinas, southernmost Texas, southern Arizona and British Columbia. Munroe (1976) recognized *Botys (Rhodaria) vinulenta* Grote & Robinson, 1867 (type locality: North America. [BMNH]) as a synonym of *Pyrausta signatalis*. In our analysis, three distinct clusters of *Pyrausta signatalis* were detected. However, the genetic distance was lower than 2.0% from each other. The morphological variation of the *Pyrausta signatalis* seems common in the eastern part of the range.

15. ***Pyrausta tuolumnalis* Barnes & McDunnough, 1918** Type locality: USA, California, Tuolumne Meadows. [USNM]

This species is similar to *Pyrausta orphisalis* Walker, 1859 (type locality: USA, Ontario, Albany R., St. Martin's Falls. [BMNH]) and ranges from the Northwest Territories, Yukon, and presumably Alaska, south through British Columbia and Alberta as far east as Lloydminster along the Saskatchewan border, and in the mountains to Los Angeles County, California, and northern New Mexico. Munroe (1976) indicated that *Pyrausta tuolumnalis* and the European *Pyrausta porphyralis* (Denis & Schiffermüller, 1775) (type locality: [Austria] Wienergegend), which is found in most Europe except Ireland, Great Britain, Portugal, Croatia, Greece and Ukraine, are morphologically nearly indistinguishable except for subtle characteristics

on the forewing. For example, he mentioned that Staudinger's record of *Pyrausta porphyralis* from Kamchatka may possibly refer to *Pyrausta tuolumnalis*. However, biologically, *Pyrauta porphyralis* is a two-generation species of moist temperate habitats, whereas *Pyrausta tuolumnalis* is northern and montane and seems to have only one generation per year. In our analyses, two distinct clusters of *Pyrausta tuolumnalis* were displayed. Interestingly, three records from Alberta and British Columbia, Canada shared identical BIN with *Pyrausta porphyralis* in BOLD. Given the short sequences of *Pyrausta tuolumnalis* from those areas, more fresh material is needed to provide reliable estimate of genetic distance and for delimiting species.

16. ***Pyrausta tyralis* (Guenée, 1854)** Type locality: Cuba

This species somewhat resembles *Pyrausta laticlavia* (Grote & Robinson, 1867) (type locality: USA, Pennsylvania. [AMNH]) and ranges from southern New York to Illinois, Florida and western Texas, south through Mexico and the West Indies, as far as Venezuela. Munroe (1976) recognized five synonyms, including two that he newly proposed : *Rhodaria agathalis* Walker, 1859 (type locality: Venezuela. [BMNH]) and *Syllythria idessa* Druce, 1895 (type locality: Guatemala, San Geronimo. [BMNH]). He also indicated that the reduced bands suffused with red in the synonym *Pyrausta erosnealis* Walker, 1859 (type locality: USA. [BMNH]). Two distinct clusters of *Pyrausta laticlavia* were displayed in our analyses. One was identified as *Pyrausta tyralis*, including records sampled from Arkansas, Illinois, Oklahoma and Florida. Another cluster includes two records from Arizona, which may represent a cryptic species that has been overlooked.

17. ***Pyrausta unifascialis* (Packard, 1873)** Type locality: USA, California

This species is very close to the European *Pyrausta aerealis* (Hübner, 1793), and perhaps is only subspecifically distinct. It has been recorded from Quebec and Maine to New York, west to the Pacific and south in the western part of the range to southern Arizona and California. Munroe (1957) recognized four subspecies of *Pyrausta unifascialis*, representing by *P. u. arizonensis* Munroe, 1957 (type locality: USA, Arizona, White Mts., Wildcat Creek. [CNC]), *P. u. rindgei* Munroe, 1957 (type locality: USA, California, Riverside Co., Rancho La Sierra near Arlington. [CNC]), *P.*

u. subolivalis (Packard, 1873) (type locality: USA, Maine) and *P. u. unifascialis* (Packard, 1873) (type locality: USA, California). In our analyses, two distinct clusters were displayed and the genetic distance was higher than 2.0% between them. One was identified as *P. u. subolivalis* including records from British Columbia, Alberta and Yukon. Another was identified as *P. u. arizonensis* and *P. u. unifascialis* including records from Arizona, California, British Columbia and Alberta. Our findings suggest that the northern subspecies *P. u. subolivalis* should be raised to species level because of morphological differences of male genitalia.

18. ***Pyrausta volupialis* (Grote, 1877)** Type locality: USA, Colorado, hills west of denver [BMNH]

This species is closely similar to *Pyrausta signatalis* (Walker, 1866) (type locality: North America. [BMNH]) in appearance and ranges from Texas, to Colorado, California, Arizona, and Mexico. Munroe (1976) indicated that *Pyrausta volupialis* is a variable species. Two distinct clusters were displayed. However, the genetic distance was lower than 2.0% between them. One includes records from Colorado, California and Arizona. Another includes a few records from Texas, New Mexico and Oklahoma besides other recorded localities in the United States.

19. ***Pyrausta zonalis* Barnes & McDunnough, 1918** Type locality: USA, California, Riverside Co., Palm Springs. [USNM]

Pyrausta zonalis is most likely to be confused with *Pyrausta napaealis* (Hulst, 1886) (type locality: USA, California [AMNH]) and *Pyrausta morenali*s (Dyar, 1908) (type locality: USA, California, Grapevine. [USNM]). Munroe (1976) indicated that *Pyrausta zonalis* is most similar to *Pyrausta napaealis* in appearance but smaller. This species not uncommon in the drier parts of southern California and occurs from Apple Valley, San Bernardino County, to Painted Gorge, Imperial County, and east to Ivanpah and Palo Verde Mountains. In our analyses, two distinct clusters of *Pyrausta zonalis* were displayed. One incudes only one record from Oso Flaco Lake, San Luis Obispo County, California, another includes remaining records San Benito County and San Diego County, California. The genetic distance was higher than 2.0% between them. Our results implied that the single specimen may represent an

overlooked species.

20. ***Sitochroa chortalis* (Grote, 1873)** Type locality: not fixed

This species is similar to its allies and often seen along with Crambinae and other moths in summer meadows and pastures by day. It has been reported from Nova Scotia to southern British Columbia and south to New Jersey, Arizona and north California. Munroe (1976) treated *Sitochroa chortalis* as a new combination transferred from the genus *Eurycreon*. But he was not able to designate a lectotype from an unknown number of syntypes described respectively in New York, Massachusetts and Alabama. In our analyses, three distinct clusters were displayed. One includes two records from New Brunswick and the second includes five records from New Brunswick and Ontario. However, the genetic distance was lower than 2.0% between them. The third cluster identified as *Sitochroa chortalis* includes a few records from Wisconsin, New Mexico, Washington, Arizona, Newfoundland and Labrador, Ontario, Saskatchewan. The genetic distances was higher than 2.0% between it and the other two clusters. This suggests that a possible cryptic species has been overlooked in eastern Canada.

References

1. Munroe EG. Pyraloidea Pyralidae comprising the subfamily Pyraustinae tribe Pyraustini (part). pp. 1-78, pls 1-4, A-H. In: Dominick RB et al., The Moths of America north of Mexico 13.2A 13.2A. London: E.W. Classey Ltd. and The Wedge Entomological Research Foundation; 1976.
2. Hulst GD. Descriptions of new Pyralidae. T Am Entomol Soc. 1886;13: 145-168. doi: 10.2307/25076474
3. ICZN. International code of zoological nomenclature. 4th ed. London: The International Trust for Zoological Nomenclature; 1999.
4. Barnes W, McDunnough JH. Check list of the Lepidoptera of boreal America. Decatur: Herald Press; 1917. pp. 1-392.
5. Hodges RW, Dominick T, Davis DR, Ferguson DC, Franclemont JG, Munroe EG, et al. Check list of the Lepidoptera of America north of Mexico including Greenland. Washington:

E.W. Classey Ltd. and The Wedge Entomological Research Foundation; 1983. pp.1-284.

6. Scholtens BG, Solis MA. Annotated check list of the Pyraloidea (Lepidoptera) of America North of Mexico, Zookeys. 2015;(535): 1-136. doi: 10.3897/zookeys.535.6086
7. McDunnough JH. A Checklist of the Lepidoptera of Canada and the United States of America. Part II: Microlepidoptera. Mem Calif Acad Sci, 1939;2: 1-171.
8. Munroe EG. North American Pyraustinae: Notes and Descriptions (Lepidoptera: Pyralidae). Can Entomol. 1951;83:161-169.
9. Barnes WM, McDunnough JH. Notes and new species. Contributions to the Natural History of the Lepidoptera of North America. 1918;4(2): 61-213.
10. Munroe EG, Becker VO, Shaffer JC, Shaffer M, Solis MA. Pyraloidea.pp. 34-105 *in* Heppner JB (ed.), Atlas of Neotropical Lepidoptera. Checklist: Part 2. Hyblaeoidea – Pyraloidea – Tortricoidea. Gainesville: Association for Tropical Lepidoptera; 1995.
11. Poole RW, Lewis RE. Nomina insecta nearctica: a check list of the insects of North America, Vol. 3: Diptera, Lepidoptera, Siphonaptera. Rockville: Entomological Information Services; 1996.
